# Redundancy between Cysteine Cathepsins in Murine Experimental Autoimmune Encephalomyelitis

**DOI:** 10.1371/journal.pone.0128945

**Published:** 2015-06-15

**Authors:** Euan Ramsay Orr Allan, Robin Michael Yates

**Affiliations:** 1 Department of Comparative Biology and Experimental Medicine, Faculty of Veterinary Medicine, University of Calgary, Alberta, Canada; 2 Department of Biochemistry and Molecular Biology, Faculty of Medicine, University of Calgary, Alberta, Canada; Uniform Services University of the Health Sciences, UNITED STATES

## Abstract

The cysteine cathepsins B, S, and L are functionally linked to antigen processing, and hence to autoimmune disorders such as multiple sclerosis. Stemming from several studies that demonstrate that mice can be protected from experimental autoimmune encephalomyelitis (EAE) through the pharmacologic inhibition of cysteine cathepsins, it has been suggested that targeting these enzymes in multiple sclerosis may be of therapeutic benefit. Utilizing mice deficient in cysteine cathepsins both individually and in combination, we found that the myelin-associated antigen myelin oligodendrocyte glycoprotein (MOG) was efficiently processed and presented by macrophages to CD4+ T cells in the individual absence of cathepsin B, S or L. Similarly, mice deficient in cathepsin B or S were susceptible to MOG-induced EAE and displayed clinical progression and immune infiltration into the CNS, similar to their wild-type counterparts. Owing to a previously described CD4+ T cell deficiency in mice deficient in cathepsin L, such mice were protected from EAE. When multiple cysteine cathepsins were simultaneously inhibited via genetic deletion of both cathepsins B and S, or by a cathepsin inhibitor (LHVS), MHC-II surface expression, MOG antigen presentation and EAE were attenuated or prevented. This study demonstrates the functional redundancy between cathepsin B, S and L in EAE, and suggests that the inhibition of multiple cysteine cathepsins may be needed to modulate autoimmune disorders such as multiple sclerosis.

## Introduction

Lysosomal cysteine cathepsins are a family of papain-like proteases that are principally charged with the hydrolysis of proteins and the activation of other proteases within endosomes and lysosomes [1–3]. These proteases are highly expressed in antigen presenting cells (APCs), and are critical for the processing of antigens as well as MHC- class II (MHC-II) invariant chain (Ii) within the endolysosomal system of these cells [3–14]. Unsurprisingly, lysosomal cysteine cathepsins (particularly cathepsin B, S, and L) have been implicated in the processing of autoantigens and thus associated with the pathogenesis of a multitude of autoimmune conditions (for review see: Vasiljeva et al. 2007) [1–3, 7, 10, 15–20].

Multiple Sclerosis (MS) is a complex inflammatory disease of the central nervous system (CNS) which is characterized by debilitating neurologic impairments that stem from chronic CNS inflammation, extensive demyelination, and lesion formation. The clinical and pathological progression of MS is driven by an autoimmune reaction of CNS-infiltrating myelin-specific autoreactive lymphocytes [21]. The initial infiltration precedes widespread activation of macrophages and microglia in and around the CNS, in parallel with continued and extensive immune infiltration of autoreactive lymphocytes and macrophages [21–23]. Experimental autoimmune encephalomyelitis (EAE) is the most commonly-used animal model for examining the immune processes associated with MS [22]. In C57BL/6 mice, EAE is a CD4+ T cell driven autoimmune disease in response to peripheral inoculation with myelin oligodendrocyte glycoprotein (MOG) protein or peptide [24]. Within the CNS, myelin debris is primarily phagocytosed by infiltrating macrophages (rather than dendritic cells or microglia) [23], proteolytically processed in the endosomal-lysosomal system, and presented on MHC-II to MOG^35-55^-specific CD4+ T cells. During this process, cysteine cathepsins are believed to be intimately involved in the processing of both the myelin-associated antigens and Ii of MHC-II. Several studies have shown that cathepsin S inhibitors can attenuate EAE and other autoimmune models, suggesting that cathepsin S may be a specific therapeutic target for MS [10, 16, 25–27]. In particular, the compound LHVS has been suggested to selectively inhibit cathepsin S and attenuate EAE [12, 16].

Despite years of interest in therapeutically targeting cysteine cathepsins in MS, and sporadic autoimmune clinical trials of lead compounds that inhibit cathepsin S [27], EAE has not been attempted in mice genetically deficient in individual cysteine cathepsins. Hence, the importance of cathepsins B, S, and L as specific therapeutic targets for MS remain unsubstantiated in an animal model. In the present study, we demonstrate that bone marrow derived macrophages (BMMØs) deficient in cathepsin B, S, or L are equally efficient at presenting MOG to CD4+ T cells. Correspondingly, we show that mice deficient in either cathepsin B or S are not clinically or immunologically protected from EAE, and that a cysteine cathepsin inhibitor (previously proposed to attenuate EAE through selective inhibition of cathepsin S) prevents EAE in a cathepsin S independent manner. Finally, we show that genetic ablation of both cathepsins B and S modulates MHC-II processing and presentation of MOG, and protects mice from EAE.

## Materials and Methods

### Mice and cells

C57BL/6 (Wild type, WT) and C57BL/6-Tg (Tcra2D2,Tcrb2D2) 1Kuch/J (2D2) mice were purchased from the Jackson Laboratory (Bar Harbor, ME, USA). (2D2 mice express a transgenic CD4+ T cell receptor (Vβ11 TCR/Vα3.2 TCR) that is specific for the I-Ab immunodominant MOG^35-55^ peptide). Mice deficient in cathepsin B [28], cathepsin S [29], cathepsin L [6], cathepsin B/S, and cathepsin S/L were provided by Dr. Yan Shi of the University of Calgary. All mice used were fully backcrossed to C57BL/6, bred in-house under identical husbandry conditions and genotyped by PCR to confirm gene deletion before use [6, 28, 29]. Animal research was performed according to protocols approved by the University of Calgary Animal Care and Use Committee (M11029) and in accordance with the Canadian Council of Animal Care. Euthanasia was conducted by CO_2_ inhalation according to approved standard operating procedures. Mice were anesthetized with a ketamine-xylazine mixture according to approved standard operating procedures. All animal work in this study was specifically approved (M11029) by the University of Calgary Animal Care and Use Committee. BMMØs were derived from bone marrow of 8–12 week old male mice using L-929-conditioned media as a source of M-CSF over a period of 10–14 days as previously described [30–34]. QPCR confirmed relative levels or absence of specific cathepsin mRNA in BMMØ cultures (S1 Protocol, S1 Fig). BMMØs were activated overnight (18 h) with recombinant IFNγ (100 U/ml, Pepro Tech) before antigen presentation assays. EAE studies were conducted using 8 and 10 week old female mice that were age matched within experiments [24, 35, 36].

### Flow cytometry

Flow cytometry data was acquired using a FACSCalibur flow cytometer (BD Biosciences, Franklin Lakes, NJ, USA) and analyzed using FLOWJO software v8.6 (Tree Star, Ashland, OR, USA). Leukocyte populations were selected using forward scatter/side scatter (FSC/SSC). All antibodies were purchased from BD Biosciences.

### Assessment of antigen processing and presentation

To determine if BMMØs from cathepsin-deficient mice are capable of presenting murine MOG antigens to 2D2 CD4+ T cells, BMMØs derived from WT and cathepsin B, S, L, B/S, or S/L deficient mice were exposed to the immunodominant I-Ab epitope murine MOG^35-55^(0, 1, 10, 25 μg/ml; 0.39–9.7 nM), or to the full extracellular domain of murine MOG (MOG^1-125^; 0, 1, 10, 25 μg/ml; 0.039–0.96 nM) [31, 34, 37, 38], for 6 h within culture medium. Cultures without BMMØs or MOG, or with concanavalin A (ConA, 5 μg/ml) treatment, were included as negative and positive controls for specific presentation and T cell proliferation respectively. MOG^35-55^ was synthesized by the University of Calgary Peptide Services, Calgary, AB, Canada. MOG^1-125^ was prepared and characterized as previously described [34]. In brief, MOG^1-125^ was cloned with a carboxy-terminal 6xHIStag, and expressed using Rosetta blue (DE3) *E*.*coli* [34]. The tagged protein was purified using Ni-NTA agarose beads (Qiagen). Endotoxin was removed using endotoxin Affisorbant agarose (polymyxin B Separapore; Bioworld, Dublin, OH, USA). Where indicated BMMØs were treated with the cathepsin inhibitor LHVS (5 μg/ml: morpholinurea-leucine-homophenylalanine-vinyl-phenyl-sulfone; PolyPeptide Laboratories, Strasbourg, France) or vehicle (dimethyl sulfoxide; DMSO) for 18 h before exposure to MOG antigens [12]. Where indicated, phorbol 12-myristate 13-acetate (PMA 50 ng/ml PMA) and ionomycin (1 μg/ml) (PI) was used as a positive control for CD4+ T cell activation. Following antigen exposure, BMMØs were washed extensively to remove all extracellular MOG antigen, and naïve 2D2 splenocytes were incubated with the BMMØs for 16 h. The presentation efficiency of MOG^35-55^ was determined by measuring the surface expression of two T cell early activation markers on the MOG^35-55^-specific CD4+ 2D2 T cells via flow cytometry according to standard practices: CD69 (the first inducible surface protein recruited during CD4+ T cell activation) and CD25 (the IL-2 receptor alpha subunit) [34, 39]. Surface expression of MHC-II (I-Ab) on BMMØs was measured by flow cytometry and represented using the mean fluorescent intensity (MFI) relative to WT control samples. Cell viability and surface expression of CD45, B7.2, and CD11b were assessed in LHVS-treated BMMØs using standard trypan blue exclusion and flow cytometric assays respectively.

### Fluorometric analysis of cysteine cathepsin activities

Fluorometric determination of cathepsins B, S and L activities was achieved by incubation of recombinant human cathepsins B, S, and L (Novoprotein, Summit, NJ, USA) with their respective specific fluorogenic substrates (Cat B: ZLR-AMC (R&D Systems, Minneapolis, MN, USA)); Cat L/S: Ac-HRYR-ACC (Calbiochem, Etobicoke, ON, Canada)) and increasing concentrations of LHVS (0.1563, 0.3125, 0.625, 1.25, 2.5, 5 ug/ml) in an assay buffer (20 mM sodium acetate pH 5.5 containing 0.675 mM KCl, 0.25 mM CaCl_2_, 0.125 mM MgCl_2_) supplemented with 500 μM L-cysteine:cystine (600:1 molar ratio) at 37°C in a FLUOstar Optima fluorescent plate reader (BMG Labtech, Ortenberg, Germany) as previously described [34, 40]. Relative hydrolytic activities were determined by calculating the slopes of the increasing substrate fluorescence (*y = mx+c*; where *y* indicates relative fluorescence, *m* indicates slope, *x* indicates time) of the initial reaction (20 min) and expressed relative to uninhibited samples.

### Induction of EAE

EAE was induced using standard protocols as previously described [24, 34, 35]. Briefly, 8–10 week old female WT and cathepsin B, S, L or B and S deficient mice were anesthetised with ketamine-xylazine. These mice were subsequently injected subcutaneously with 200 μl of an emulsion of 50 μg MOG^35-55^ in complete Freund’s adjuvant (0.5 mg/ml *M*. *butyricum* in paraffin oil) (CFA) (BD Difco, Franklin Lakes NJ, USA). Pertussis toxin (PT) (300 ng) (List Biological laboratories Inc., Campbell CA, USA) was injected intraperitoneally (I.P.) (pH = 7, in saline) on days 0 and day 2. Mice were clinically scored and weighed daily for 40 days using a standard scoring system (score 0—asymptomatic; 0.5—tail weakness; 1—limp tail; 1.5—hind limb limping; 2—hind limb weakness; 2.5—partial hind limb paralysis; 3—complete hind limb paralysis; 3.5—hind limb paralysis with forelimb weakness; 4—forelimb paralysis; 4.5–5—complete morbidity/death) [24, 34]. Mice were euthanized if they reached a clinical score of 4. A second group of mice was sacrificed 15 days post injection and their spinal cords were removed for analysis by flow cytometry [24, 34, 41]. Mice injected with CFA and PT in the absence of MOG^35-55^ did not develop clinical symptoms of EAE (S2 Fig). Where indicated, LHVS (25 mg/kg in 10 μl) or vehicle (DMSO) was injected I.P. daily, starting on the day of MOG immunization, in WT and cathepsin S deficient mice [16]. These mice were sacrificed at day 18 for analysis of spinal cord-infiltrating leukocytes.

### EAE spinal cord leukocyte analysis and immune profiling

Spinal cord-infiltrating and resident leukocytes were isolated from mouse spinal cords 15 or 18 days post injection with MOG^35-55^ using a discontinuous Percoll gradient as previously described [34, 36]. Cells were enumerated using a haemocytometer before being immunostained for macrophages (CD11b+/CD45+ high), microglia (CD11b+/CD45+ low), CD4+ T cells (CD4+/CD3+), CD8+ T cells (CD8+/CD3+), and B cells (B220+/CD45+) and analyzed by flow cytometry [34]. Cells were gated based on live leukocytes followed by their respective cell marker/s and represented as absolute numbers (a function of proportion of each cell type and total number of cells isolated for that tissue) as previously described [34] (S3 Fig). To compare CD4+ T cell development in naïve WT, cathepsin L deficient and cathepsin B/S deficient mice, absolute numbers of CD8+ and CD4+ T cells were enumerated in the spleen, inguinal lymph nodes (LN) and thymus as described for the spinal cord tissue above (S3 Fig) [6].

### Statistics

Statistical analyses were completed by one-way ANOVA (or unpaired Student’s t test) with a Tukey test (p<0.05). Analyses of clinical data were done using a Kruskal-Wallis test (Dunn’s multiple comparisons) of mean clinical score for each day, in addition to the total area under the curve [34]. Statistical analyses were completed using GraphPad Prism software (La Jolla, CA, USA).

## Results

### BMMØs deficient in cysteine cathepsin B, S, or L can efficiently process and present MOG via MHC-II

**Fig 1:**
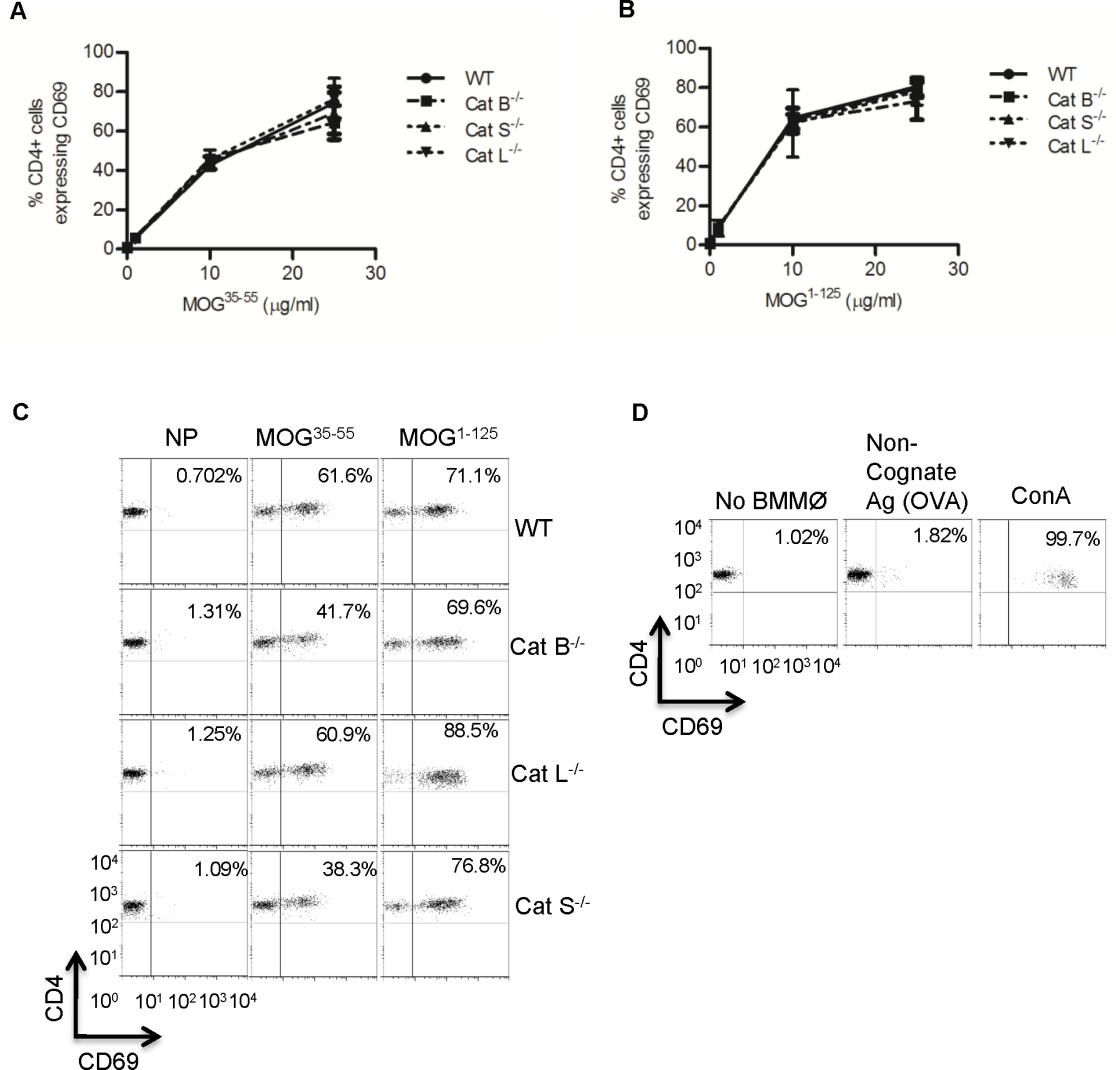
BMMØs derived from WT mice and mice deficient in cathepsin B, S, or L process and present MHC-II-restricted MOG antigens equivalently. WT, cathepsin B (Cat B^-/-^), cathepsin S (Cat S^-/-^), cathepsin L (Cat L^-/-^) deficient BMMØ were incubated for 6 h with (A) MOG^35-55^ peptide (0, 1, 10, 25 μg/ml) or (B) MOG^1-125^ (0, 1, 10, 25 μg/ml). Activation of MOG^35-55^-specific 2D2 CD4+ T cells was determined by surface expression of CD69 after 16 h exposure to the BMMØs. Data represent 4 independent experiments. Data presented as mean +/- SEM (ANOVA, p<0.05); significant differences from internal WT controls are denoted by asterisks (*). (C,D) Representative flow cytometry plots of samples containing 25 μg/ml MOG^35-55^ and MOG^1-125^, and control samples that contain no BMMØs, a non-cognate antigen (ovalbumin, OVA) and the T cell mitogen concanavalin A (ConA 5 μg/ml).

Since it has been shown that cysteine cathepsins are involved in MHC-II restricted antigen presentation, and that macrophages are the primary APC responsible CD4+ T cell reactivation in the CNS during EAE [21, 23], we set to determine if BMMØs deficient in cathepsin B, S, or L could efficiently process and present MHC-II-restricted MOG antigens. Specifically we measured the ability of BMMØs deficient in individual cysteine cathepsins, to process and present the I-Ab immunodominant MOG^35-55^ epitope to MOG^35-55^-specfic 2D2 transgenic CD4+ T cells. In brief, BMMØs were incubated for 6 h with the pre-processed MOG^35-55^ peptide or the whole antigen MOG^1-125^, and subsequently co-incubated with 2D2 CD4+ T cells for 16 h. Activation of 2D2 CD4+T cells was assessed by examining surface marker CD69, which is an indicator of early T cell activation [34, 39, 42, 43]. When pulsed with the pre-processed MOG^35-55^ peptide antigen, BMMØs deficient in cathepsin B, S, or L activated the MOG^35-55^-specfic 2D2 CD4+ T cells as efficiently as WT BMMØs, indicating that these mice are equally capable of presenting pre-processed peptide in the context of MHC-II (Fig 1A). Similarly, when these BMMØs were incubated with the unprocessed antigen MOG^1-125^, they stimulated equivalent levels of CD69 on 2D2 CD4+ T cells, indicating no overt deficiency in MOG processing in the absence of one of these cysteine proteases (Fig 1B–1D). Similar results were obtained using CD25 as an alternative marker of T cell activation (S4 Fig).

### Mice deficient in either cathepsin B or S are susceptible to MOG-induced EAE, while mice deficient in cathepsin L are protected from EAE due to a systemic CD4+ T cell deficiency

**Fig 2:**
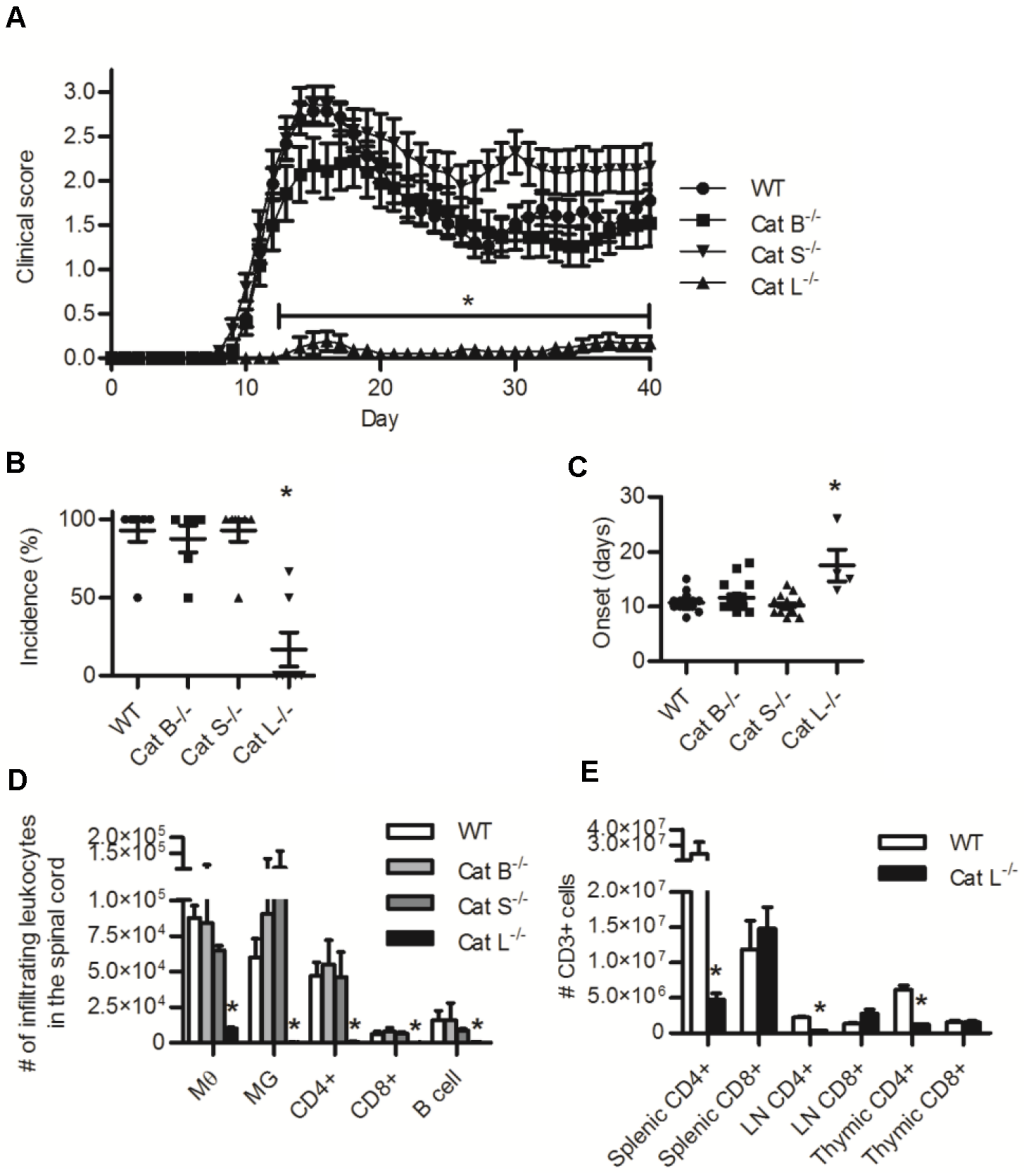
Mice deficient in cathepsin B or S are susceptible to MOG^35-55^ induced EAE while mice deficient in cathepsin L are protected from EAE and have a systemic deficiency of CD4+ T cells. Induction of EAE was attempted in WT, cathepsin B (Cat B^-/-^), cathepsin S (Cat S^-/-^), and cathepsin L (Cat L^-/-^) deficient mice by inoculation of 50μg MOG^35-55^ in CFA (day 0) and 300 ng pertussis toxin (day 0 and 2). (A) Clinical scoring of EAE severity over 40 days post inoculation (N = 20–21 mice). (B) Clinical incidence (percentage of mice that exceeded a clinical score of 0). (C) Number of days to clinical onset (time between inoculation and a clinical score of at least 0.5). (D) Absolute numbers of macrophages (MØ, CD11b+/CD45 high), microglia (MG, CD11b+/CD45 low), CD4+ T cells (CD4+/CD3+), CD8+ T cells (CD8+/CD3+) and B cells (B220+/CD45+) isolated from the spinal cord (via a discontinuous percoll gradient) of mice 15 days following inoculation with MOG^35-55^ (n = 3–4). (E) The abundance of CD3+/CD4+ and CD3+/CD8+ cells in the spleen, lymph nodes, and thymus from WT and cathepsin L (Cat L^-/-^) deficient mice. Data presented as mean+/- SEM; significant differences (Clinical data, Kruskal-Wallis of daily clinical scores and total area under the curve; ANOVA or unpaired students t-test, p<0.05) from the WT internal control are denoted by asterisks (*).

Given that BMMØs deficient in cathepsin B, S, or L can process and present MOG^35-55^ from MOG^1-125^, we assessed the susceptibility of mice deficient in these cathepsins to MOG-driven EAE. As anticipated, EAE elicited in mice deficient in cathepsins B or S was not clinically (Fig 2A–2C) or immunologically (Fig 2D, S3 Fig) distinguishable from EAE in WT mice. Mice deficient in cathepsin L however were almost completely protected from EAE. Mice deficient in cathepsin L had a significantly reduced clinical score (Fig 2A), 4 fold lower incidence (Fig 2B), and a five day delay (Fig 2C) when compared to WT and cathepsin B or S deficient mice. This correlated with an approximate 10 fold reduction in macrophages, microglia, CD4+ T cells, CD8+ T cells, and B cells isolated from the spinal cords of mice that were sacrificed at day 15 post-inoculation (Fig 2D). Since cathepsin L has been previously shown to be critical for positive thymic selection of CD4+ T cells, we enumerated T cells within the thymus, spleen, and LNs of uninoculated cathepsin L-deficient mice [6]. As previously reported, we found that mice deficient in cathepsin L, but not mice deficient in cathepsin B or S, had significantly reduced numbers of CD4+ T cells in these lymphoid tissues (Fig 2E, S3 Fig) [6]. Since EAE in C57BL/6 mice is primarily mediated by CD4+ T cells, defective thymic selection in these mice is most likely responsible for the observed protection from EAE. Nevertheless, the observation that mice deficient in cathepsin B or S are susceptible to EAE led us to revisit the previously observed protection from EAE by specific pharmacological inhibition of these individual enzymes.

### LHVS treatment prevents efficient MHC-II restricted antigen presentation of MOG antigens, and prevents development of EAE in a cathepsin S independent manner

**Fig 3:**
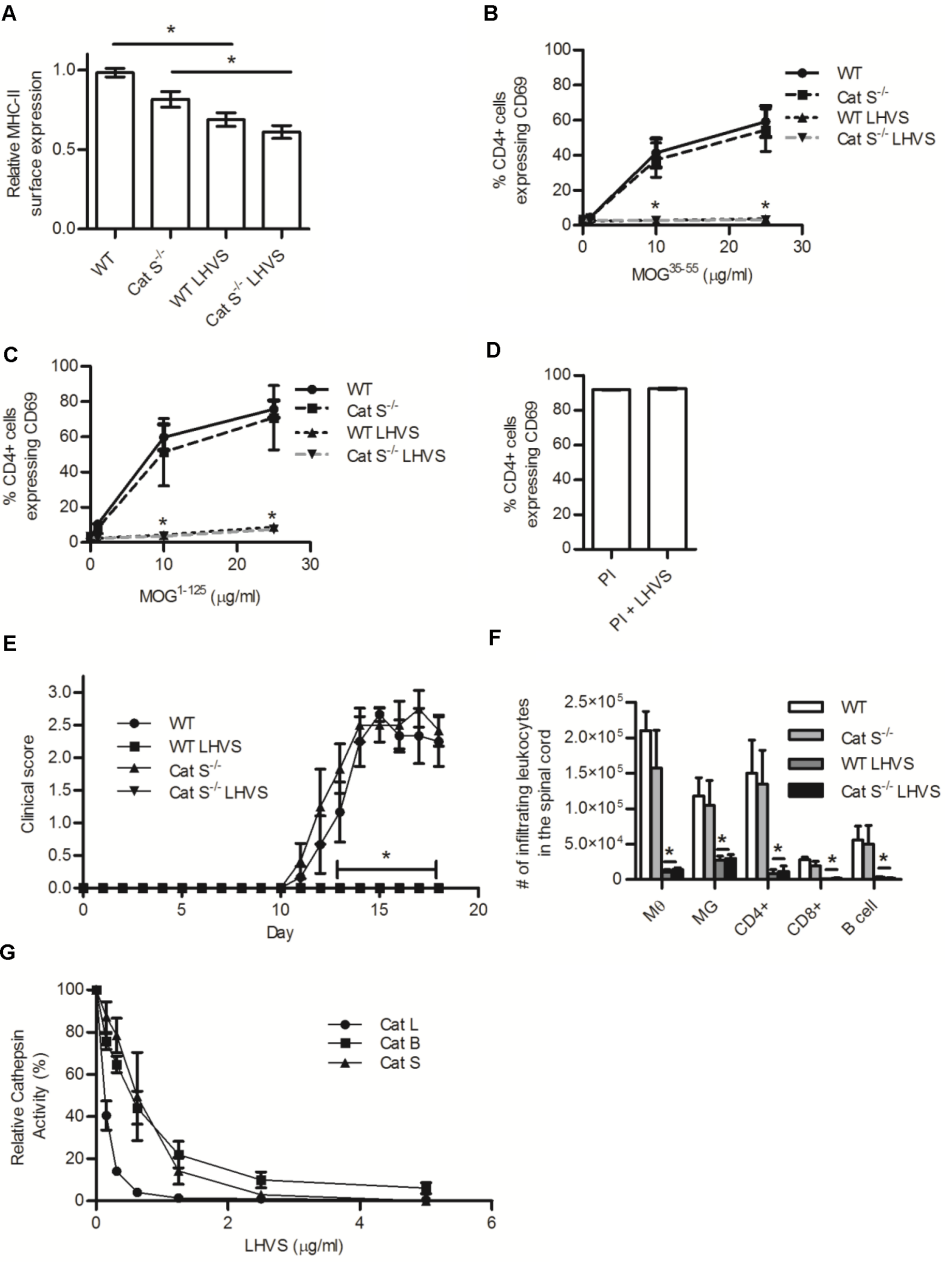
LHVS treatment reduces MHC-II surface expression and MHC-II restricted presentation efficiencies of MOG antigens in BMMØ, and protects from EAE in WT and cathepsin S deficient mice. (A-C) WT and cathepsin S (Cat S^-/-^) deficient BMMØ were treated with LHVS (5μg/ml) or DMSO (carrier) for 18 h before assessment of (A) MHC-II surface expression (I-Ab quantified by MFI) or their ability to activate MOG^35-55^-specific CD4+ T cells following incubation with (B) MOG^35-55^ peptide (0, 1, 10, 25 μg/ml), (C) MOG^1-125^ (0, 1, 10, 25 μg/ml), or (D) PMA (50 ng/ml) and ionomysin (1 μg/ml) (PI). Activation of MOG^35-55^-specific 2D2 CD4+ T cells was determined by surface expression of CD69 after 16 h incubation with the BMMØ (n = 4). (E) Clinical disease course of WT and Cat S^-/-^ deficient mice after induction of EAE with 50μg MOG^35-55^ in CFA (day 0) and 300 ng pertussis toxin (day 0 and 2) with daily injection of 25 mg/kg LHVS or DMSO (carrier) (day 0–15) (n = 6). (F) Absolute numbers of macrophages (MØ, CD11b+/CD45 high), microglia (MG, CD11b+/CD45 low), CD4+ T cells (CD4+/CD3+), CD8+ T cells (CD8+/CD3+), and B cells (B220+/CD45+) isolated from the spinal cord (via a discontinuous percoll gradient) of mice 18 days following inoculation with MOG^35-55^ (n = 4). (G) The relative enzymatic activity of recombinant cathepsins B, S, and L in the presence of increasing concentrations of LHVS (n = 3). Relative activities were determined by the rate of increase in fluorescence of cathepsin-selective fluorogenic substrates at 37°C, pH 5.5, and expressed relative to uninhibited samples. Data presented as mean+/- SEM; significant differences (Clinical data, Kruskal-Wallis; ANOVA, p<0.05) from the WT internal control are denoted by asterisks (*).

It has been previously reported that pharmacological inhibition of cathepsin S by compounds such as LHVS, compromises the efficiency of antigen presentation of ovalbumin and protects mice from MOG-induced EAE [12, 16]. Since we found that mice deficient in cathepsin S are susceptible to EAE and have APCs that can efficiently present MOG antigens, we set to reconcile these findings by investigating the effect of LHVS, on MOG processing and susceptibility to EAE in WT and cathepsin S deficient mice. BMMØs derived from WT and cathepsin S deficient mice were pre-incubated with LHVS (or vehicle) and assessed for MHC-II surface levels as well as their ability to process and present MOG to 2D2 CD4+ T cells. LHVS treatment of BMMØs significantly reduced their surface expression of MHC-II without affecting surface expression of B7.2, CD45, or CD11b or viability (Fig 3A; S5 Fig). Additionally, LHVS treatment of BMMØs ablated their ability to present either processed (MOG^35-55^) or unprocessed (MOG^1-125^) antigen, in a cathepsin S independent manner (Fig 3B and 3C; S4 Fig,S6 Fig). LHVS did not directly impact T cell activation in response to PI (Fig 3D). Treatment of the BMMØs with the cysteine cathepsin inhibitors E-64d and leupeptin also attenuated efficiencies of MOG presentation (S7 Fig). Consistent with previous reports, daily injections of LHVS completely prevented the clinical onset of EAE in WT mice (Fig 3E and 3F) [16]. Intriguingly, mice deficient in cathepsin S were equally protected from EAE by LHVS treatment, suggesting that LHVS protection is either independent of cathepsin S, or that LHVS-mediated inhibition of multiple cysteine cathepsins is necessary for protection from EAE. Indeed, in reconstituted assays we found that LHVS inhibited the activities of cysteine cathepsins B and L as efficiently as cathepsins S (Fig 3G).

### Deficiency of both cathepsins B and S reduces MHC-II restricted presentation of MOG antigens and prevents development of EAE

**Fig 4:**
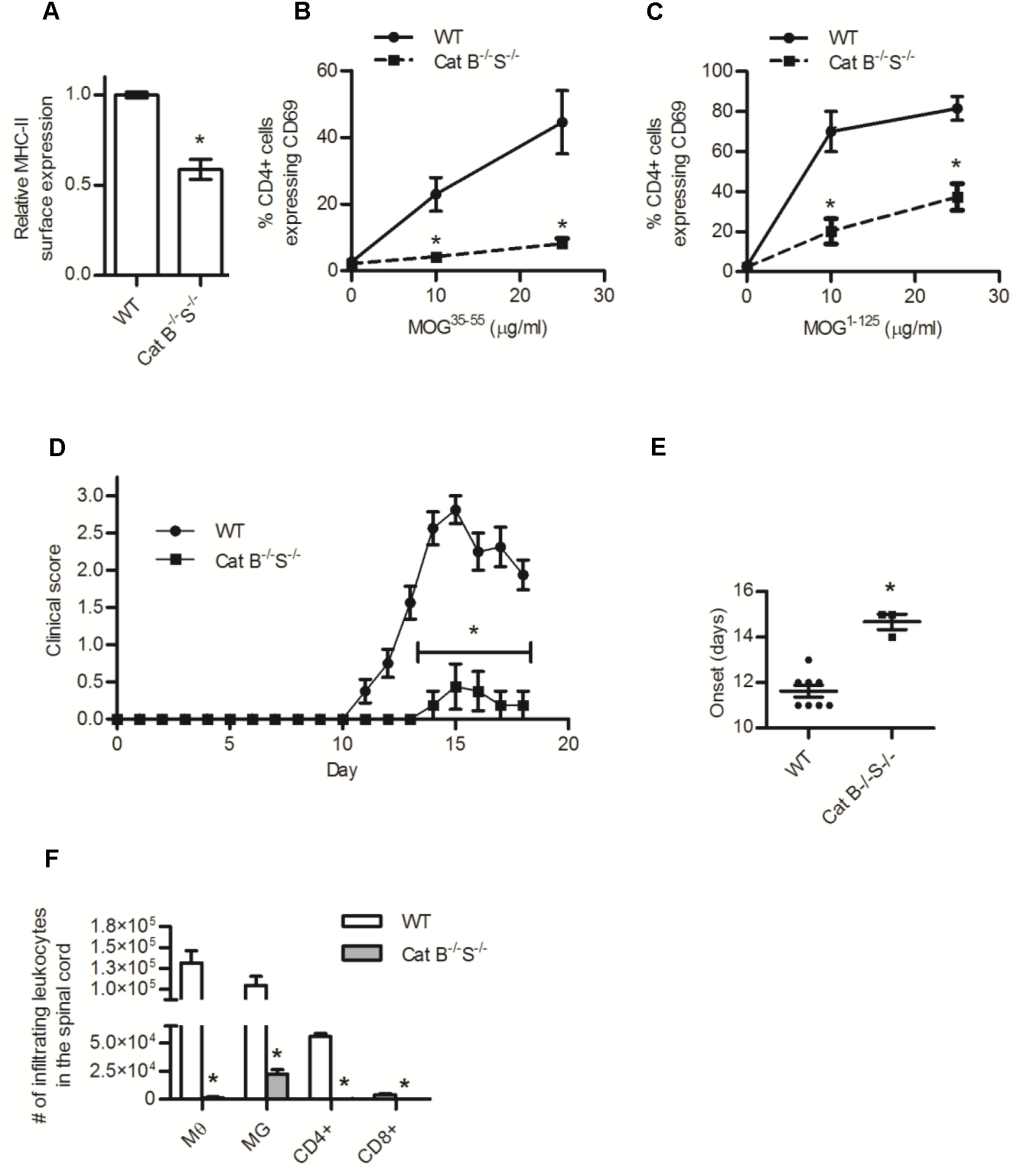
BMMØ deficient in both cathepsins B and S have reduced MHC-II surface expression and presentation of MOG antigens, and mice deficient in both cathepsins B and S are protected from EAE. (A-C) WT and cathepsin B and S (Cat B^-/-^S^-/-^) deficient BMMØ were (A) examined for their MHC-II surface expression (I-Ab, quantified by MFI), or their ability to activate MOG^35-55^-specific CD4+ T cells following incubation with (B) MOG^35-55^ peptide (0, 10, 25 μg/ml) or (C) MOG^1-125^ (0, 10, 25 μg/ml). Activation of MOG^35-55^-specific 2D2 CD4+ T cells was determined by surface expression of CD69 after 16 h incubation with the BMMØ (n = 4). (D) Clinical disease course and (E) number of days to clinical onset (score of at least 0.5) of WT and Cat B^-/-^S^-/-^ deficient mice after active induction of EAE with 50μg MOG^35-55^ in CFA (day 0) and 300 ng pertussis toxin (day 0 and 2), (N = 8). (F) The number of macrophages (MØ, CD11b+/CD45 high), microglia (MG, CD11b+/CD45 low), CD4+ T cells (CD4+/CD3+), and CD8+ T cells (CD8+/CD3+) isolated from the spinal cord (via a discontinuous percoll gradient) of mice 18 days following inoculation with MOG^35-55^ (n = 3). Data presented as mean+/- SEM; significant differences (Clinical data, Mann-Whitney Test; unpaired students t-testp<0.05) from the WT internal control are denoted by asterisks (*).

To determine whether presentation of MOG and susceptibility to EAE would be modulated by the absence of multiple cysteine cathepsins, we generated mice deficient in both cathepsin S and cathepsin B. BMMØs derived from these mice (cathepsin B^-/-^S^-/-^) had significantly lower surface expression of MHC-II and reduced abilities to present MOG antigens to 2D2 CD4+ T cells (Fig 4A–4C, S4 Fig, S6 Fig). Accordingly, the cathepsin B^-/-^S^-/-^ mice were protected from EAE, exhibiting lower clinical scores, delayed onset of disease and fewer infiltrating leukocytes isolated from spinal cord tissue (Fig 4D–4F). Since the absence of cathepsin L leads to overt CD4+ T cells deficiencies, EAE was not explored in mice deficient in both cathepsin S and cathepsin L, however BMMØs derived from cathepsin S^-/-^L^-/-^ mice displayed similarly reduced MOG presentation efficiencies as those derived from cathepsin B^-/-^S^-/-^ mice (S8 Fig). Together these findings indicate that mice are able to present MHC-II antigens and generate a robust CD4+ T cell immune response in the absence of cathepsin S, but not when combined with the absence of other cysteine cathepsins.

## Discussion

This work adds to the growing collection of studies that demonstrate a functional redundancy between the lysosomal cysteine cathepsins B, S and L with respect to antigen processing, antigen presentation, and autoimmunity [11, 34]. While previous studies have explored pharmacologic inhibition of cysteine cathepsins in murine EAE, this is the first study to directly examine the roles of individual cathepsins in MOG processing and EAE by utilizing mice genetically deficient in specific cysteine cathepsins. In this study BMMØs derived from mice deficient in cathepsins B, S, or L were capable of efficiently presenting the I-Ab-immunodominant MOG epitope, and mice deficient in cathepsin B or S were fully susceptible to EAE. Unsurprisingly, mice deficient in cathepsin L were protected from EAE presumably owing to the previously-described thymic immunodeficiency [6]. We additionally demonstrated that LHVS, a reportedly selective inhibitor of cathepsin S, protected mice from EAE but did so independently of cathepsin S [12, 16]. Deficiencies in both cathepsins B and cathepsin S led to significantly reduced MHC-II expression and MOG antigen presentation efficiency, and protection from EAE. Although the effect of cathepsin deficiency/inhibition on the efficiency of MOG/MHC-II processing and presentation correlated with EAE susceptibility in this study, it is possible that alteration of other cathepsin-dependent processes contributed to the EAE phenotypes observed. Nonetheless, these findings suggest that the inhibition of multiple cysteine cathepsins (via broad spectrum pharmacologic inhibition) may be necessary to attenuate certain autoimmune diseases.

Although mice deficient in cathepsins B or S generally have functional immune systems, several studies have suggested non-redundant roles for cathepsin S in the context of particular models of autoimmunity [7, 10, 12, 15, 16]. Changes in cathepsin B activity have also been implicated in numerous neurologic autoimmune diseases [19, 44–46]. In the context of MOG-induced EAE in C57BL/6 (I-Ab) mice, we found that the ablation of cathepsin B activity has no effect on the EAE pathogenesis or MOG antigen processing and presentation by macrophages [19, 44–46]. These results support the previous findings that splenocytes deficient in cathepsin B are still capable of presenting antigens via MHC-II [28]. Unlike cathepsin B, cathepsin S has been shown to be necessary for efficient presentation of a variety of antigens in DCs and B cells which do not typically express cathepsin L, [4, 7, 11, 20, 34, 47, 48]. Correspondingly cathepsin S deficient mice were protected from collagen induced arthritis (CIA), which requires robust B cell and DC responses for clinical progression [7, 49]. In contrast to CIA, it has been shown that macrophages are the key APCs for reactivation of CD4+ T cells during demyelination in the CNS of C57BL/6 mice in EAE [11, 21, 23]. Since cathepsin L and S share similar cleavage sites and are coexpressed by macrophages, unlike B cells and DCs, it is possible that cathepsin L activity compensates for the absence of cathepsin S in these critical APCs for EAE [7, 11, 34]. Indeed, we demonstrated that BMMØs deficient in either cathepsin S or cathepsin L can efficiently process and present MOG in I-Ab, but not in the absence of both enzymes. Moreover, we showed that loss of cathepsin B in addition to cathepsin S also compromised antigen presentation. Together these data suggest that cathepsin B and cathepsin L are both necessary, but not individually sufficient, to compensate for the loss of cathepsin S in macrophages. This was supported by the observations that mice deficient in either cathepsin S or cathepsin B, but not both, were susceptible to EAE.

LHVS, among other cathepsin S inhibitors, has been shown to attenuate EAE [12, 16]. Our results demonstrate that LHVS, although being commonly referred to as a selective cathepsin S inhibitor [12, 16, 50], can efficiently inhibit cathepsin B and L. Indeed, LHVS treatment of macrophages lowered MHC-II surface expression and abolished MOG antigen presentation independently of cathepsin S. Unsurprisingly LHVS protected both WT and cathepsin S-deficient mice from EAE [16]. These findings suggest that the specific inhibition of cathepsin S by LHVS is insufficient to protect from EAE, and that the compound’s inhibition of multiple cysteine cathepsins is necessary for its therapeutic action.

Cathepsin S has been considered a potential therapeutic target in human autoimmune disorders such as MS for over 30 years [7, 10, 16, 26, 51]. Despite this interest, numerous autoimmune clinical trials targeting cathepsin S have failed to show clinical efficacy, and patent applications for cathepsin S inhibitors have declined in the last decade [27]. Our findings, derived from the analysis of cathepsin deficient mice, suggest that targeting individual cysteine cathepsins, such as cathepsin S, may not be sufficient to inhibit macrophage-driven autoimmune processes, and that the functional redundancies between cysteine cathepsins need to be considered for future therapeutic strategies targeting these enzymes.

## Supporting Information

S1 Fig Conformation of cathepsin deficiency in BMMØs. Cathepsin B, S, and L mRNA levels in WT, cathepsin B (Cat B^-/-^), cathepsin S (Cat S^-/-^), cathepsin L (Cat L^-/-^) deficient BMMØs. Average mRNA expression levels quantified by QPCR, are presented relative to WT controls (n = 3). mRNA levels normalized to 18S. Data presented as mean +/- SEM (ANOVA, p<0.05); significant differences from WT BMMØs are denoted by asterisks (*).
(PPTX)

S2 Fig Mice inoculated with CFA/PBS/PT do not exhibit clinical or immunological symptoms of EAE. Clinical scoring of EAE severity after inoculation of WT mice with 50μg MOG^35-55^ in CFA (WT) or PBS in CFA (WT mock) (day 0) and 300 ng pertussis toxin (day 0 and 2) for 40 days (N = 9). Numbers of macrophages (MØ, CD11b+/CD45 high), microglia (MG, CD11b+/CD45 low), CD4+ T cells (CD4+/CD3+), CD8+ T cells (CD8+/CD3+) and B cells (B220+/CD45+) isolated from the spinal cord (via a discontinuous percoll gradient) of mice 15 days following inoculation with MOG^35-55^ (n = 3). Data presented as mean+/- SEM; significant differences (Clinical data, Kruskal-Wallis; unpaired students t-test, p<0.05) from the WT internal control are denoted by asterisks (*).
(PPTX)

S3 Fig Flow cytometry gating strategies for cells isolated from the spinal cord, and thymus, spleen, and inguinal lymph node (LN). Infiltrating and resident leukocytes were isolated from mouse spinal cords 15 or 18 days post injection with MOG^35-55^ using a discontinuous Percoll gradient. Cells were counted with a haemocytometer before being immunostained for markers of macrophages (CD11b+/CD45+ high), microglia (CD11b+/CD45+ low), CD4+ T cells (CD4+/CD3+), CD8+ T cells (CD8+/CD3+) and B cells (B220+/CD45+), and analyzed by flow cytometry. To compare lymphocyte development between WT, cathepsin L- and cathepsin B/S-deficient mice, leukocytes were isolated from spleens, lymph nodes and thymuses of naïve mice using a discontinuous Percoll gradient and immunostained for markers of CD4+ T cells (CD4+/CD3+) and CD8+ T cells (CD8+/CD3+) and analyzed by flow cytometry.
(PPTX)

S4 Fig Analysis of antigen presentation using CD25 as an alternative marker for CD4+ T cell activation generated equivalent results to those found via evaluation of CD69 expression. WT, WT LHVS treated, cathepsin B (Cat B^-/-^), cathepsin S (Cat S^-/-^), cathepsin L (Cat L^-/-^), or cathepsin B and S (Cat B^-/-^S^-/-^) deficient BMMØ were incubated for 6 h with MOG^35-55^ peptide (0, 1, 10, 25 μg/ml) or MOG^1-125^ (0, 1, 10, 25 μg/ml). Activation of MOG^35-55^-specific 2D2 CD4+ T cells was determined by surface expression of CD25 after 16 h exposure to the BMMØs. Representative flow cytometry plots for WT, cathepsin B (Cat B^-/-^), cathepsin S (Cat S^-/-^), cathepsin L (Cat L^-/-^) deficient BMMØ incubated with MOG^35-55^ (25 μg/ml) or MOG^1-125^ (25 μg/ml). Data represent 3 independent experiments. Data presented as mean +/- SEM (ANOVA, p<0.05); significant differences from internal WT controls are denoted by asterisks (*).
(PPTX)

S5 Fig BMMØ treated with 5 μg/ml LHVS do not exhibit modified cell survival, or surface expression of CD11b, CD45, or B7.2. Cells were exposed to LHVS for 24 h (5 μg/ml unless otherwise indicated) and assessed for cell viability by trypan blue exclusion using standard protocols. Surface expression of CD11b, CD45, and B7.2 were analyzed by flow cytometry following immunostaining of the treated BMMØs. Data represent 3 independent experiments. Data presented as mean +/- SEM (ANOVA, p<0.05); significant differences from internal WT controls are denoted by asterisks (*).
(PPTX)

S6 Fig Representative flow cytometry plots for Figs 3 and 4. Activation of MOG^35-55^-specific 2D2 CD4+ T cells was determined by surface expression of CD69 after 16 h incubation with WT, LHVS-treated or cathepsin B^-/-^S^-/-^ BMMØ that had been previously pulsed with 25 μg/ml MOG^35-55^ or no peptide (NP).
(PPTX)

S7 Fig Inhibition of cysteine cathepsins reduces MHC-II restricted presentation efficiencies of MOG antigens in BMMØ. WT BMMØ were treated overnight with DMSO vehicle (untreated), E-64d (10 μg/ml) and leupeptin (2.5 μg/ml) and were subsequently incubated for 6 h with MOG^35-55^ peptide (0, 10, 25 μg/ml) or MOG^1-125^ (0, 10, 25 μg/ml). Activation of MOG^35-55^-specific 2D2 CD4+ T cells was determined by surface expression of CD69 after 16 h exposure to the pulsed and washed BMMØs. Representative flow cytometry plots of no peptide (NP), or 25 μg/ml MOG^35-55^. Percentage of live BMMØ (as evaluated by trypan blue exclusion) after 24 h exposure to E-64d (10 μg/ml) and Leupeptin (2.5 μg/ml). Data represent 3 independent experiments. Data presented as mean +/- SEM (ANOVA, p<0.05); significant differences from internal WT controls are denoted by asterisks (*).
(PPTX)

S8 Fig BMMØ deficient in both cathepsin S and L have reduced MHC-II restricted presentation efficiencies of MOG antigens. BMMØ derived from WT mice and mice deficient in both cathepsin S and L (Cat S^-/-^L^-/-^) were examined for their ability to activate MOG^35-55^-specific CD4+ T cells following incubation with MOG^35-55^ peptide (0, 10, 25 μg/ml) or MOG^1-125^ (0, 10, 25 μg/ml). Activation of MOG^35-55^-specific 2D2 CD4+ T cells was determined by surface expression of CD69 after exposure to the pulsed and washed BMMØs. Data represent 4 independent experiments. Data presented as mean+/- SEM; significant differences (unpaired students t-test, p<0.05) from the WT internal control are denoted by asterisks (*).
(PPTX)

S1 Protocol RNA extraction, cDNA synthesis and real-time polymerase chain reaction (QPCR). Methods for RNA extraction, cDNA synthesis, and QPCR of cathepsin mRNA levels in WT and cathepsin deficient BMMØs shown in S1 Fig.
(DOCX)
