# Supplementary material for: Redundancy between Cysteine Cathepsins in Murine Experimental Autoimmune Encephalomyelitis
Source: PLoS One. 2015 Jun 15;10(6):e0128945. doi: 10.1371/journal.pone.0128945 (PMC4468166; doi:10.1371/journal.pone.0128945)
Supplement: S1 Fig — Cathepsin B, S, and L mRNA levels in WT, cathepsin B (Cat B-/-), cathepsin S (Cat S-/-), cathepsin L (Cat L-/-) deficient BMMØs. Average mRNA expression levels quantified by QPCR, are presented relative to WT controls (n = 3). mRNA levels normalized to 18S. Data presented as mean +/- SEM (ANOVA, p<0.05); significant differences from WT BMMØs are denoted by asterisks (*). (PPTX) [file pone.0128945.s001.pptx]

## Slide 1
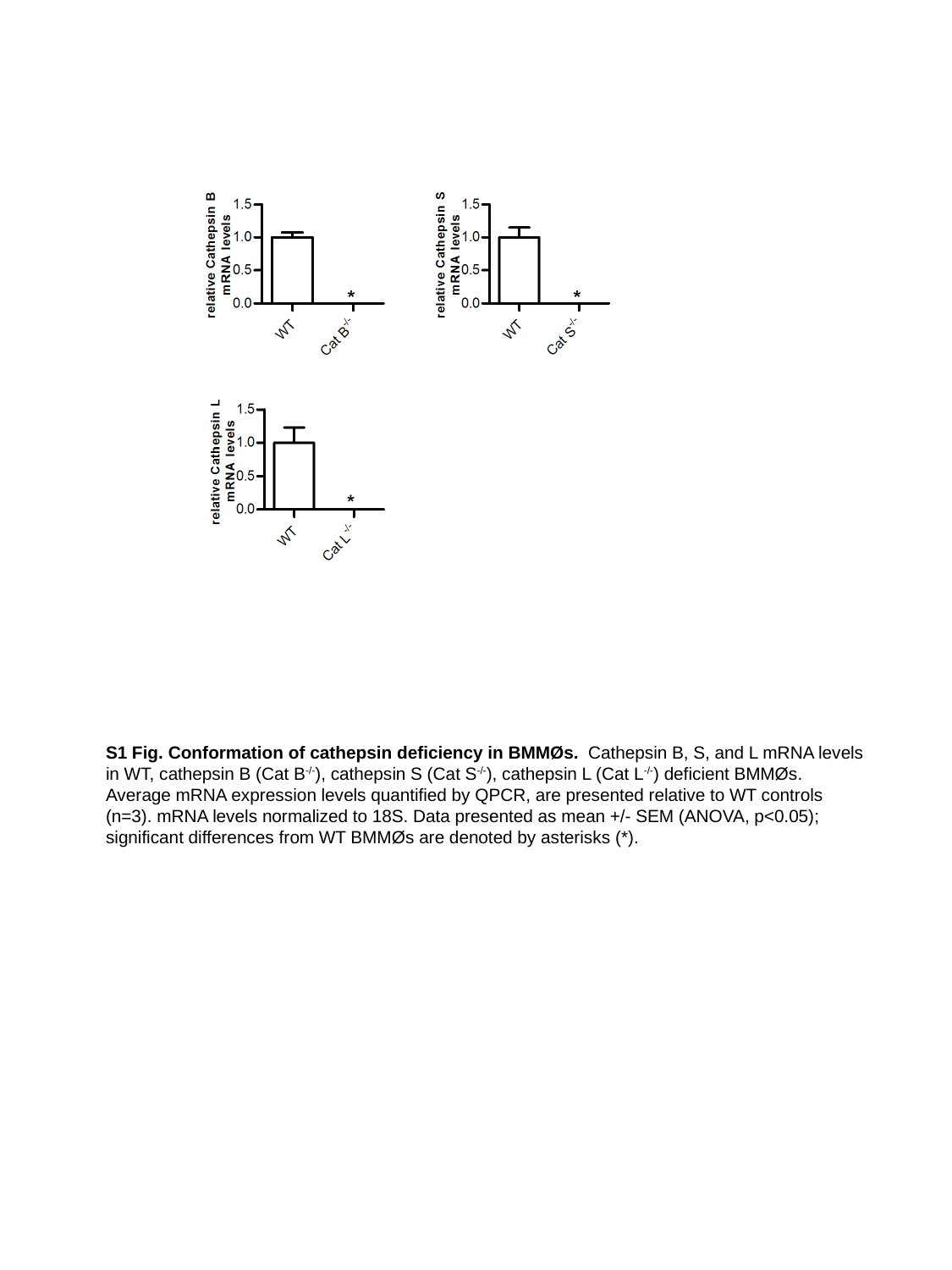

S1 Fig. Conformation of cathepsin deficiency in BMMØs. Cathepsin B, S, and L mRNA levels in WT, cathepsin B (Cat B-/-), cathepsin S (Cat S-/-), cathepsin L (Cat L-/-) deficient BMMØs. Average mRNA expression levels quantified by QPCR, are presented relative to WT controls (n=3). mRNA levels normalized to 18S. Data presented as mean +/- SEM (ANOVA, p<0.05); significant differences from WT BMMØs are denoted by asterisks (*).
