# Supplementary material for: Redundancy between Cysteine Cathepsins in Murine Experimental Autoimmune Encephalomyelitis
Source: PLoS One. 2015 Jun 15;10(6):e0128945. doi: 10.1371/journal.pone.0128945 (PMC4468166; doi:10.1371/journal.pone.0128945)
Supplement: S2 Fig — Clinical scoring of EAE severity after inoculation of WT mice with 50μg MOG35-55 in CFA (WT) or PBS in CFA (WT mock) (day 0) and 300 ng pertussis toxin (day 0 and 2) for 40 days (N = 9). Numbers of macrophages (MØ, CD11b+/CD45 high), microglia (MG, CD11b+/CD45 low), CD4+ T cells (CD4+/CD3+), CD8+ T cells (CD8+/CD3+) and B cells (B220+/CD45+) isolated from the spinal cord (via a discontinuous percoll gradient) of mice 15 days following inoculation with MOG35-55 (n = 3). Data presented as mean+/- SEM; significant differences (Clinical data, Kruskal-Wallis; unpaired students t-test, p<0.05) from the WT internal control are denoted by asterisks (*). (PPTX) [file pone.0128945.s002.pptx]

## Slide 1
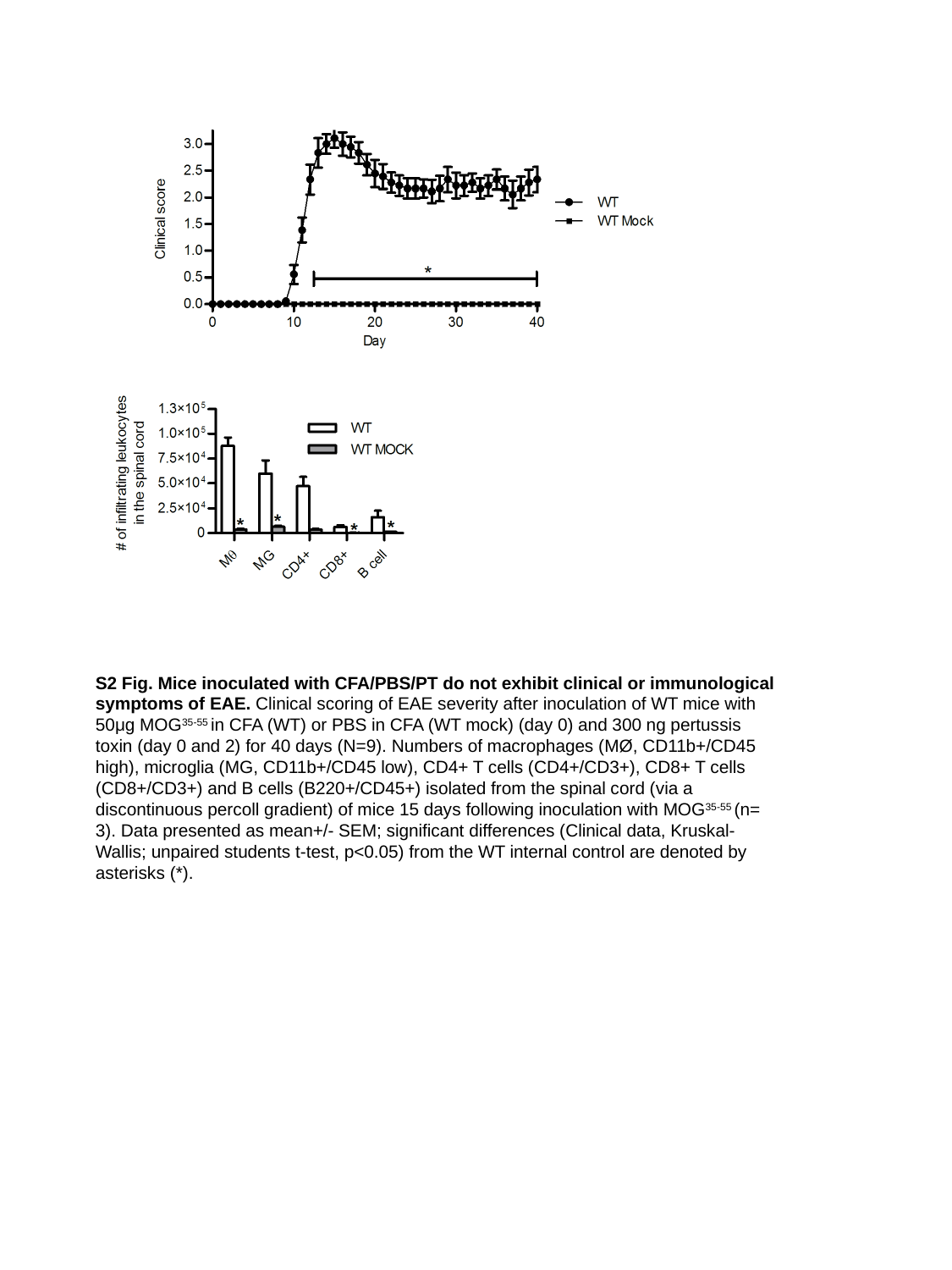

S2 Fig. Mice inoculated with CFA/PBS/PT do not exhibit clinical or immunological symptoms of EAE. Clinical scoring of EAE severity after inoculation of WT mice with 50μg MOG35-55 in CFA (WT) or PBS in CFA (WT mock) (day 0) and 300 ng pertussis toxin (day 0 and 2) for 40 days (N=9). Numbers of macrophages (MØ, CD11b+/CD45 high), microglia (MG, CD11b+/CD45 low), CD4+ T cells (CD4+/CD3+), CD8+ T cells (CD8+/CD3+) and B cells (B220+/CD45+) isolated from the spinal cord (via a discontinuous percoll gradient) of mice 15 days following inoculation with MOG35-55 (n= 3). Data presented as mean+/- SEM; significant differences (Clinical data, Kruskal-Wallis; unpaired students t-test, p<0.05) from the WT internal control are denoted by asterisks (*).
