# Supplementary material for: Redundancy between Cysteine Cathepsins in Murine Experimental Autoimmune Encephalomyelitis
Source: PLoS One. 2015 Jun 15;10(6):e0128945. doi: 10.1371/journal.pone.0128945 (PMC4468166; doi:10.1371/journal.pone.0128945)
Supplement: S3 Fig — Infiltrating and resident leukocytes were isolated from mouse spinal cords 15 or 18 days post injection with MOG35-55 using a discontinuous Percoll gradient. Cells were counted with a haemocytometer before being immunostained for markers of macrophages (CD11b+/CD45+ high), microglia (CD11b+/CD45+ low), CD4+ T cells (CD4+/CD3+), CD8+ T cells (CD8+/CD3+) and B cells (B220+/CD45+), and analyzed by flow cytometry. To compare lymphocyte development between WT, cathepsin L- and cathepsin B/S-deficient mice, leukocytes were isolated from spleens, lymph nodes and thymuses of naïve mice using a discontinuous Percoll gradient and immunostained for markers of CD4+ T cells (CD4+/CD3+) and CD8+ T cells (CD8+/CD3+) and analyzed by flow cytometry. (PPTX) [file pone.0128945.s003.pptx]

## Slide 1
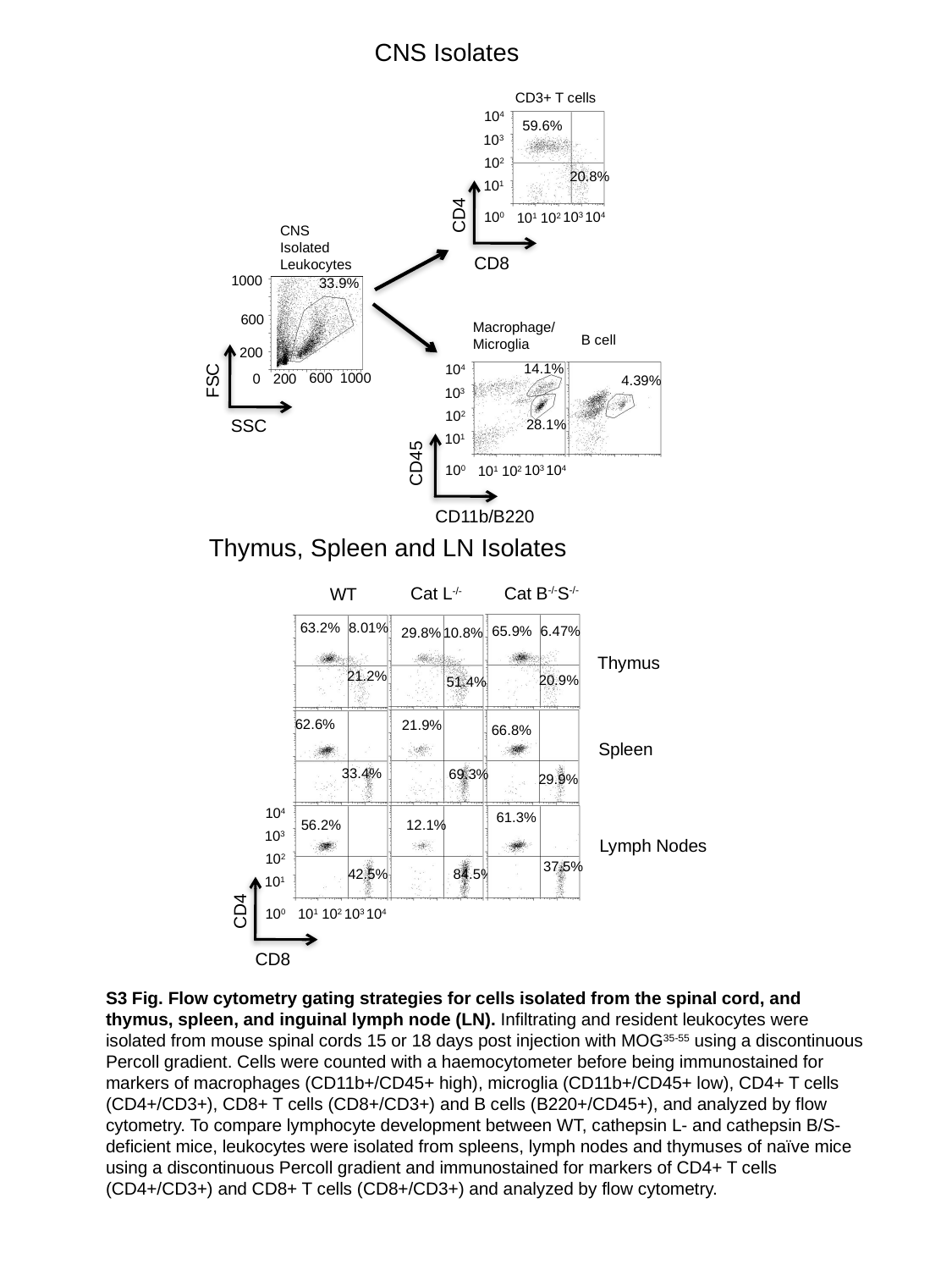

CNS Isolates
CD3+ T cells
104
59.6%
103
102
20.8%
101
CD4
104
103
100
102
101
CD8
1000
33.9%
600
200
FSC
1000
600
0
200
SSC
Macrophage/Microglia
B cell
14.1%
104
4.39%
103
102
28.1%
101
CD45
104
103
100
102
101
CD11b/B220
CNS Isolated
Leukocytes
Thymus, Spleen and LN Isolates
Cat B-/-S-/-
Cat L-/-
WT
63.2%
8.01%
65.9%
6.47%
29.8%
10.8%
21.2%
20.9%
51.4%
62.6%
21.9%
66.8%
33.4%
69.3%
29.9%
104
12.1%
56.2%
103
Lymph Nodes
102
84.5%
42.5%
101
CD4
104
103
100
102
101
CD8
Thymus
Spleen
61.3%
37.5%
S3 Fig. Flow cytometry gating strategies for cells isolated from the spinal cord, and thymus, spleen, and inguinal lymph node (LN). Infiltrating and resident leukocytes were isolated from mouse spinal cords 15 or 18 days post injection with MOG35-55 using a discontinuous Percoll gradient. Cells were counted with a haemocytometer before being immunostained for markers of macrophages (CD11b+/CD45+ high), microglia (CD11b+/CD45+ low), CD4+ T cells (CD4+/CD3+), CD8+ T cells (CD8+/CD3+) and B cells (B220+/CD45+), and analyzed by flow cytometry. To compare lymphocyte development between WT, cathepsin L- and cathepsin B/S-deficient mice, leukocytes were isolated from spleens, lymph nodes and thymuses of naïve mice using a discontinuous Percoll gradient and immunostained for markers of CD4+ T cells (CD4+/CD3+) and CD8+ T cells (CD8+/CD3+) and analyzed by flow cytometry.
