# Supplementary material for: Redundancy between Cysteine Cathepsins in Murine Experimental Autoimmune Encephalomyelitis
Source: PLoS One. 2015 Jun 15;10(6):e0128945. doi: 10.1371/journal.pone.0128945 (PMC4468166; doi:10.1371/journal.pone.0128945)
Supplement: S4 Fig — WT, WT LHVS treated, cathepsin B (Cat B-/-), cathepsin S (Cat S-/-), cathepsin L (Cat L-/-), or cathepsin B and S (Cat B-/-S-/-) deficient BMMØ were incubated for 6 h with MOG35-55 peptide (0, 1, 10, 25 μg/ml) or MOG1-125 (0, 1, 10, 25 μg/ml). Activation of MOG35-55-specific 2D2 CD4+ T cells was determined by surface expression of CD25 after 16 h exposure to the BMMØs. Representative flow cytometry plots for WT, cathepsin B (Cat B-/-), cathepsin S (Cat S-/-), cathepsin L (Cat L-/-) deficient BMMØ incubated with MOG35-55 (25 μg/ml) or MOG1-125 (25 μg/ml). Data represent 3 independent experiments. Data presented as mean +/- SEM (ANOVA, p<0.05); significant differences from internal WT controls are denoted by asterisks (*). (PPTX) [file pone.0128945.s004.pptx]

## Slide 1
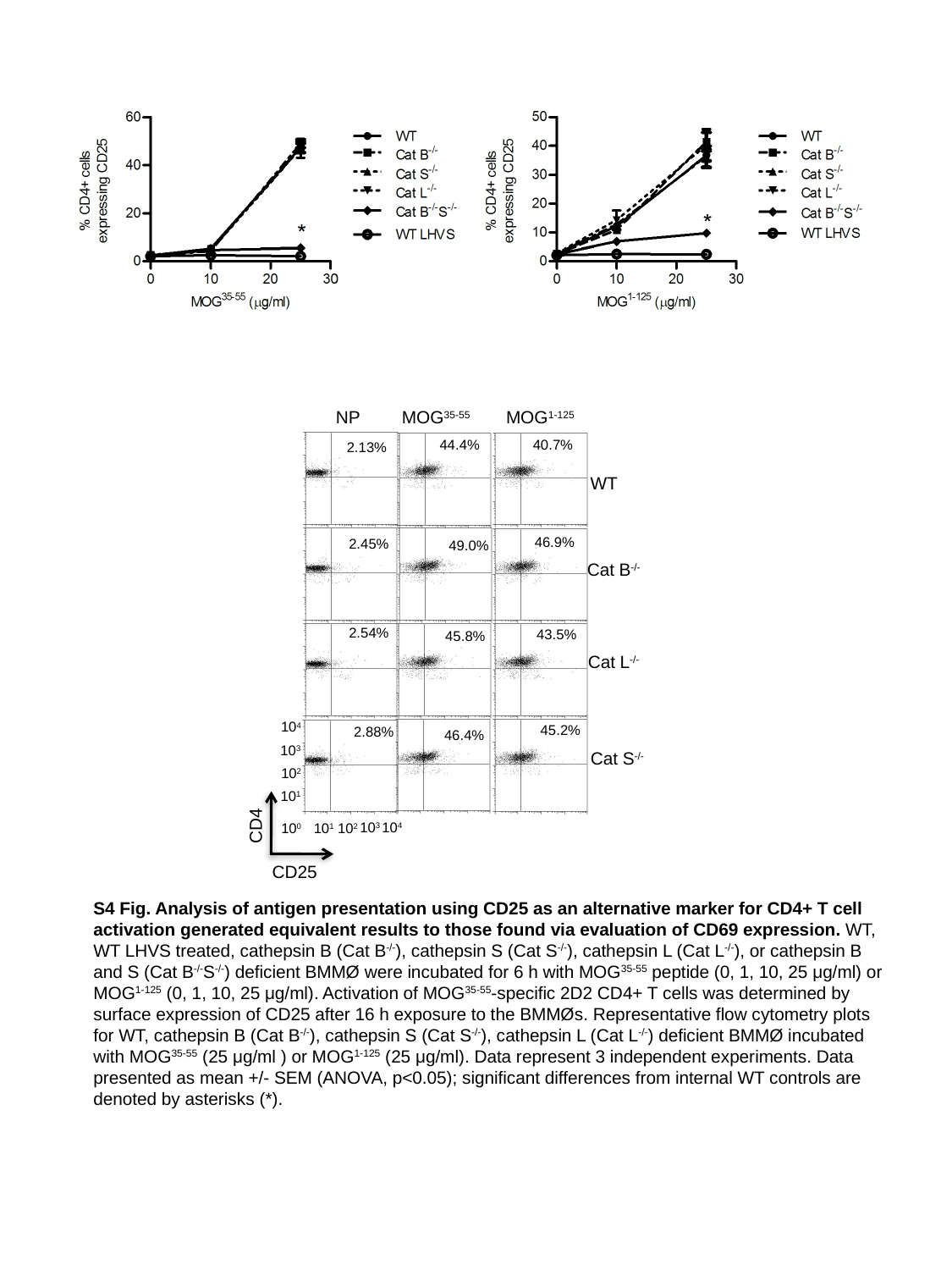

MOG35-55
MOG1-125
NP
40.7%
44.4%
2.13%
WT
46.9%
2.45%
49.0%
Cat B-/-
2.54%
43.5%
45.8%
Cat L-/-
104
45.2%
2.88%
46.4%
103
Cat S-/-
102
101
CD4
104
103
100
102
101
CD25
S4 Fig. Analysis of antigen presentation using CD25 as an alternative marker for CD4+ T cell activation generated equivalent results to those found via evaluation of CD69 expression. WT, WT LHVS treated, cathepsin B (Cat B-/-), cathepsin S (Cat S-/-), cathepsin L (Cat L-/-), or cathepsin B and S (Cat B-/-S-/-) deficient BMMØ were incubated for 6 h with MOG35-55 peptide (0, 1, 10, 25 μg/ml) or MOG1-125 (0, 1, 10, 25 μg/ml). Activation of MOG35-55-specific 2D2 CD4+ T cells was determined by surface expression of CD25 after 16 h exposure to the BMMØs. Representative flow cytometry plots for WT, cathepsin B (Cat B-/-), cathepsin S (Cat S-/-), cathepsin L (Cat L-/-) deficient BMMØ incubated with MOG35-55 (25 μg/ml ) or MOG1-125 (25 μg/ml). Data represent 3 independent experiments. Data presented as mean +/- SEM (ANOVA, p<0.05); significant differences from internal WT controls are denoted by asterisks (*).
