# Supplementary material for: Redundancy between Cysteine Cathepsins in Murine Experimental Autoimmune Encephalomyelitis
Source: PLoS One. 2015 Jun 15;10(6):e0128945. doi: 10.1371/journal.pone.0128945 (PMC4468166; doi:10.1371/journal.pone.0128945)
Supplement: S5 Fig — Cells were exposed to LHVS for 24 h (5 μg/ml unless otherwise indicated) and assessed for cell viability by trypan blue exclusion using standard protocols. Surface expression of CD11b, CD45, and B7.2 were analyzed by flow cytometry following immunostaining of the treated BMMØs. Data represent 3 independent experiments. Data presented as mean +/- SEM (ANOVA, p<0.05); significant differences from internal WT controls are denoted by asterisks (*). (PPTX) [file pone.0128945.s005.pptx]

## Slide 1
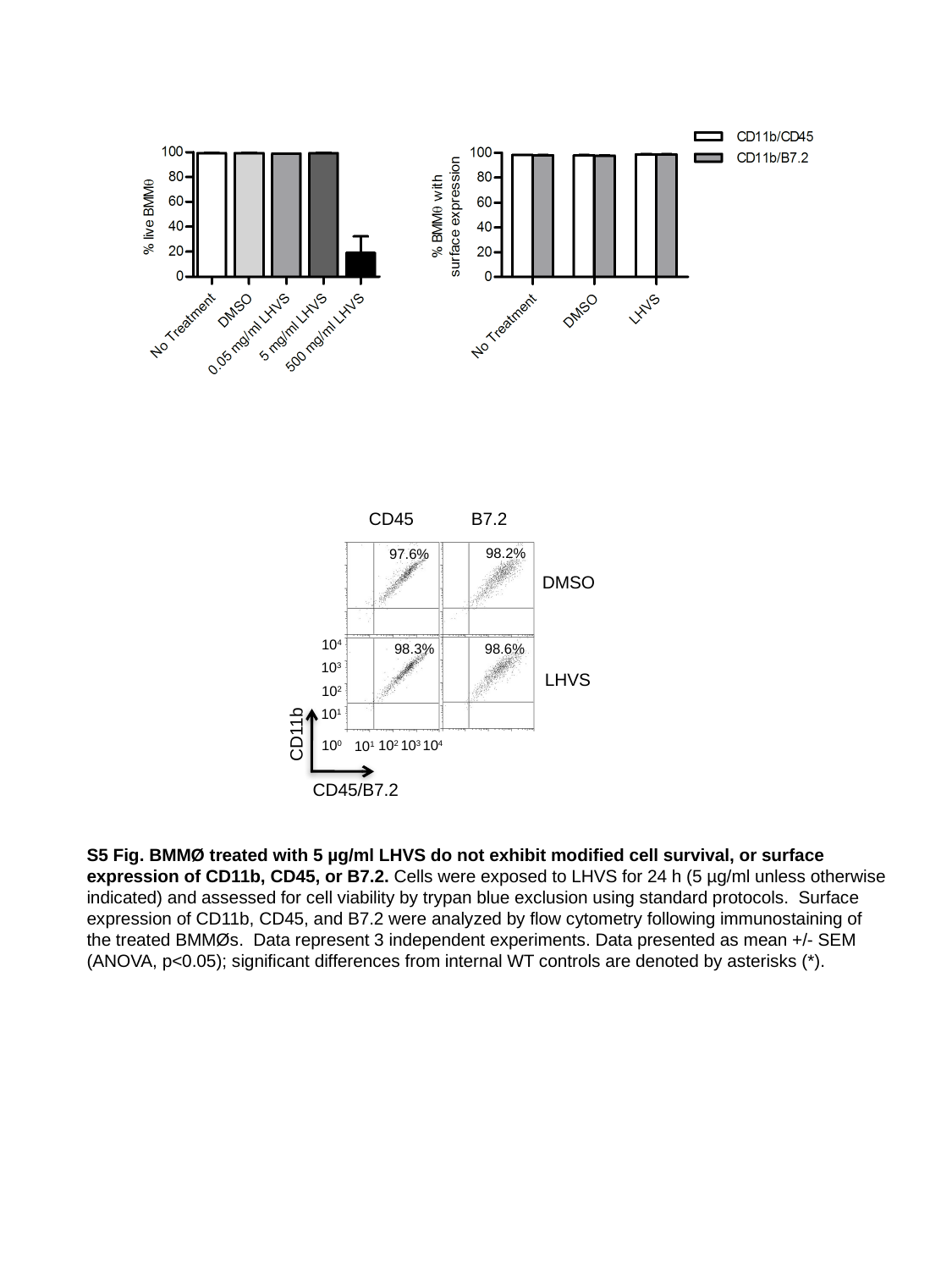

B7.2
CD45
98.2%
97.6%
DMSO
104
98.3%
98.6%
103
LHVS
102
101
CD11b
104
103
100
102
101
CD45/B7.2
S5 Fig. BMMØ treated with 5 µg/ml LHVS do not exhibit modified cell survival, or surface expression of CD11b, CD45, or B7.2. Cells were exposed to LHVS for 24 h (5 µg/ml unless otherwise indicated) and assessed for cell viability by trypan blue exclusion using standard protocols. Surface expression of CD11b, CD45, and B7.2 were analyzed by flow cytometry following immunostaining of the treated BMMØs. Data represent 3 independent experiments. Data presented as mean +/- SEM (ANOVA, p<0.05); significant differences from internal WT controls are denoted by asterisks (*).
