# Supplementary material for: Redundancy between Cysteine Cathepsins in Murine Experimental Autoimmune Encephalomyelitis
Source: PLoS One. 2015 Jun 15;10(6):e0128945. doi: 10.1371/journal.pone.0128945 (PMC4468166; doi:10.1371/journal.pone.0128945)
Supplement: S6 Fig — Activation of MOG35-55-specific 2D2 CD4+ T cells was determined by surface expression of CD69 after 16 h incubation with WT, LHVS-treated or cathepsin B-/-S-/- BMMØ that had been previously pulsed with 25 μg/ml MOG35-55 or no peptide (NP). (PPTX) [file pone.0128945.s006.pptx]

## Slide 1
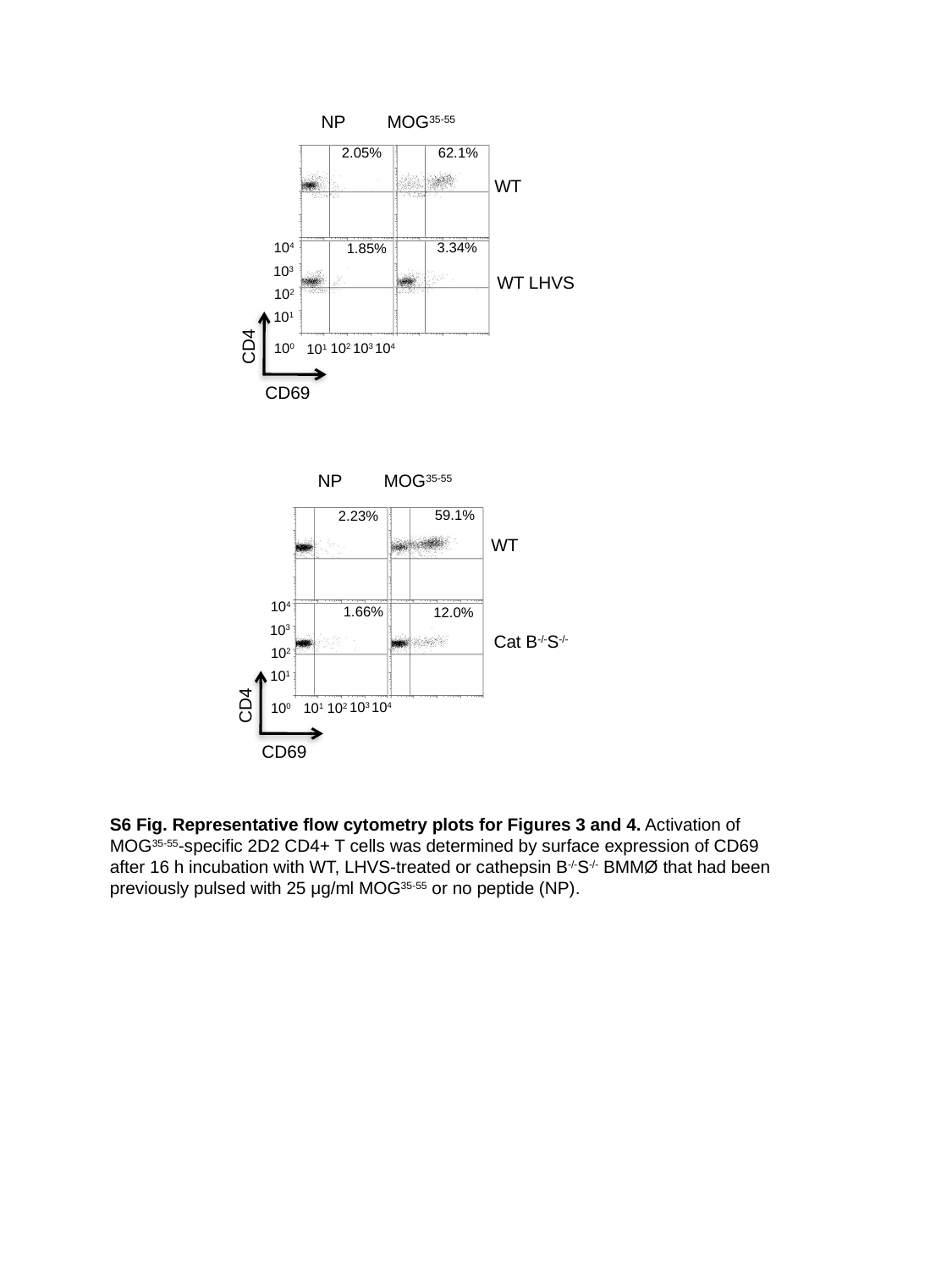

MOG35-55
NP
62.1%
2.05%
WT
104
3.34%
1.85%
103
WT LHVS
102
101
CD4
104
103
100
102
101
CD69
MOG35-55
NP
59.1%
2.23%
WT
104
1.66%
12.0%
103
Cat B-/-S-/-
102
101
CD4
104
103
100
102
101
CD69
S6 Fig. Representative flow cytometry plots for Figures 3 and 4. Activation of MOG35-55-specific 2D2 CD4+ T cells was determined by surface expression of CD69 after 16 h incubation with WT, LHVS-treated or cathepsin B-/-S-/- BMMØ that had been previously pulsed with 25 μg/ml MOG35-55 or no peptide (NP).
