# Supplementary material for: Redundancy between Cysteine Cathepsins in Murine Experimental Autoimmune Encephalomyelitis
Source: PLoS One. 2015 Jun 15;10(6):e0128945. doi: 10.1371/journal.pone.0128945 (PMC4468166; doi:10.1371/journal.pone.0128945)
Supplement: S7 Fig — WT BMMØ were treated overnight with DMSO vehicle (untreated), E-64d (10 μg/ml) and leupeptin (2.5 μg/ml) and were subsequently incubated for 6 h with MOG35-55 peptide (0, 10, 25 μg/ml) or MOG1-125 (0, 10, 25 μg/ml). Activation of MOG35-55-specific 2D2 CD4+ T cells was determined by surface expression of CD69 after 16 h exposure to the pulsed and washed BMMØs. Representative flow cytometry plots of no peptide (NP), or 25 μg/ml MOG35-55. Percentage of live BMMØ (as evaluated by trypan blue exclusion) after 24 h exposure to E-64d (10 μg/ml) and Leupeptin (2.5 μg/ml). Data represent 3 independent experiments. Data presented as mean +/- SEM (ANOVA, p<0.05); significant differences from internal WT controls are denoted by asterisks (*). (PPTX) [file pone.0128945.s007.pptx]

## Slide 1
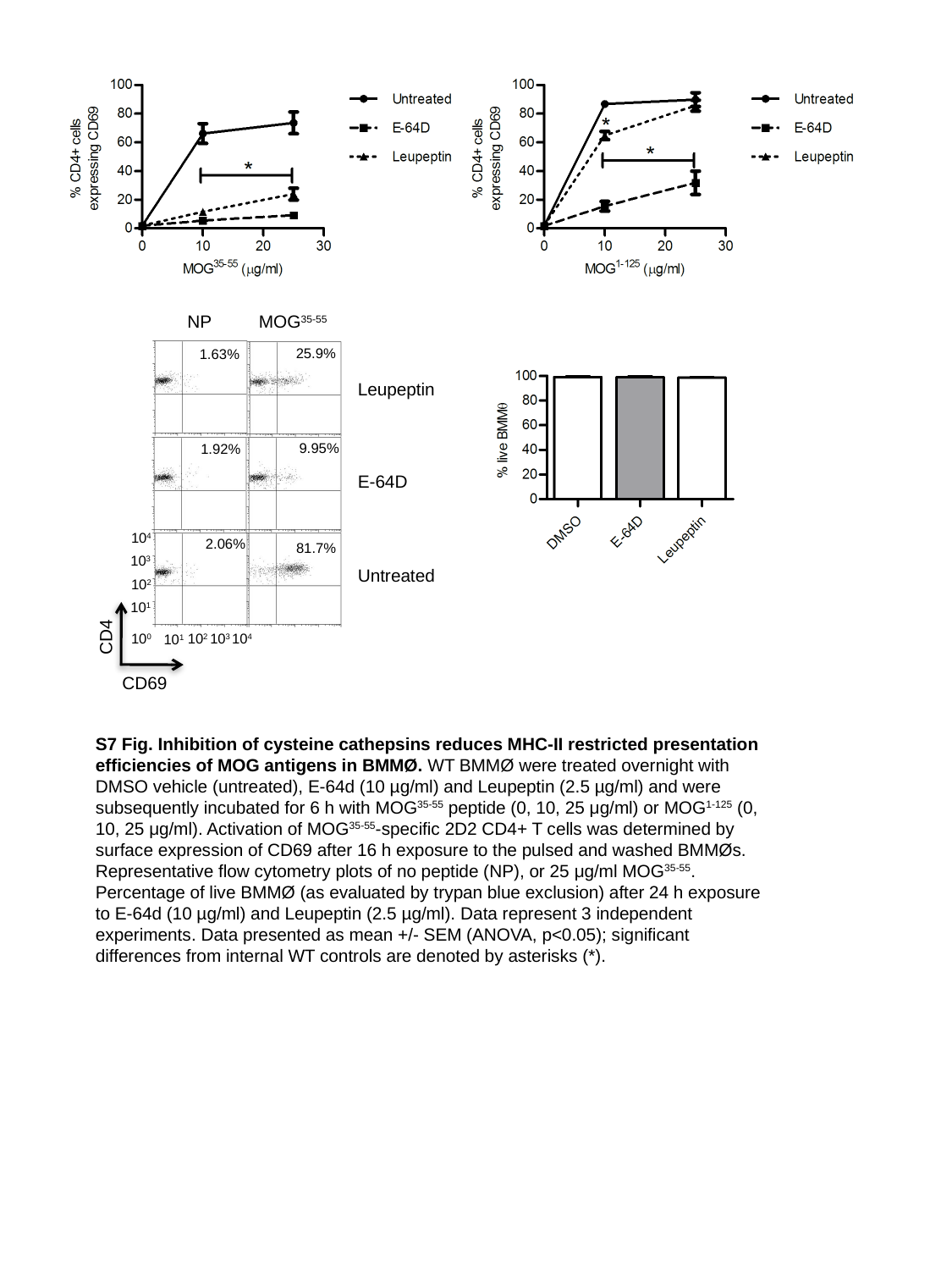

MOG35-55
NP
25.9%
1.63%
Leupeptin
9.95%
1.92%
E-64D
104
2.06%
81.7%
103
Untreated
102
101
CD4
104
103
100
102
101
CD69
S7 Fig. Inhibition of cysteine cathepsins reduces MHC-II restricted presentation efficiencies of MOG antigens in BMMØ. WT BMMØ were treated overnight with DMSO vehicle (untreated), E-64d (10 µg/ml) and Leupeptin (2.5 µg/ml) and were subsequently incubated for 6 h with MOG35-55 peptide (0, 10, 25 μg/ml) or MOG1-125 (0, 10, 25 μg/ml). Activation of MOG35-55-specific 2D2 CD4+ T cells was determined by surface expression of CD69 after 16 h exposure to the pulsed and washed BMMØs. Representative flow cytometry plots of no peptide (NP), or 25 μg/ml MOG35-55. Percentage of live BMMØ (as evaluated by trypan blue exclusion) after 24 h exposure to E-64d (10 µg/ml) and Leupeptin (2.5 µg/ml). Data represent 3 independent experiments. Data presented as mean +/- SEM (ANOVA, p<0.05); significant differences from internal WT controls are denoted by asterisks (*).
