# Supplementary material for: Redundancy between Cysteine Cathepsins in Murine Experimental Autoimmune Encephalomyelitis
Source: PLoS One. 2015 Jun 15;10(6):e0128945. doi: 10.1371/journal.pone.0128945 (PMC4468166; doi:10.1371/journal.pone.0128945)
Supplement: S8 Fig — BMMØ derived from WT mice and mice deficient in both cathepsin S and L (Cat S-/-L-/-) were examined for their ability to activate MOG35-55-specific CD4+ T cells following incubation with MOG35-55 peptide (0, 10, 25 μg/ml) or MOG1-125 (0, 10, 25 μg/ml). Activation of MOG35-55-specific 2D2 CD4+ T cells was determined by surface expression of CD69 after exposure to the pulsed and washed BMMØs. Data represent 4 independent experiments. Data presented as mean+/- SEM; significant differences (unpaired students t-test, p<0.05) from the WT internal control are denoted by asterisks (*). (PPTX) [file pone.0128945.s008.pptx]

## Slide 1
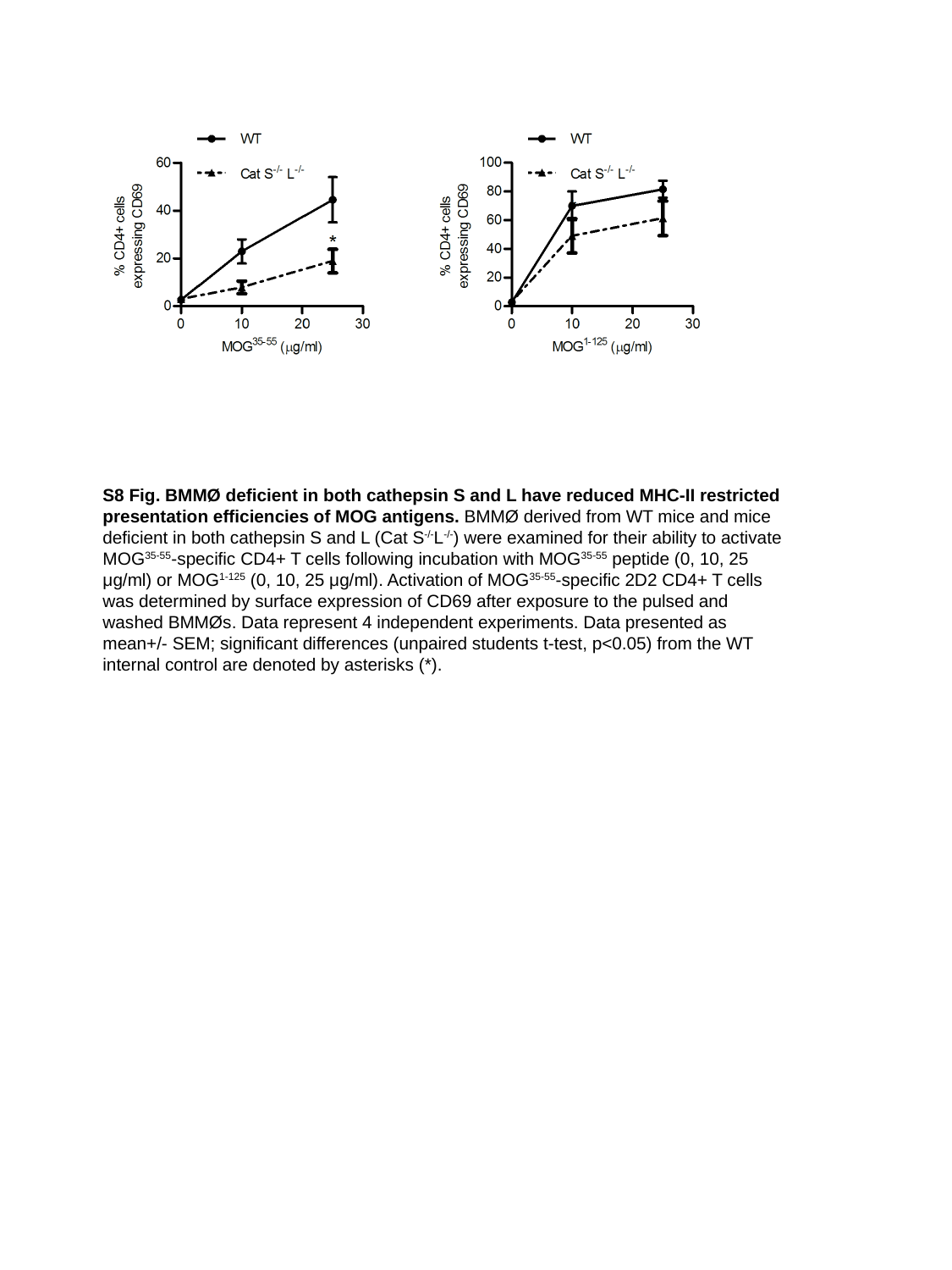

S8 Fig. BMMØ deficient in both cathepsin S and L have reduced MHC-II restricted presentation efficiencies of MOG antigens. BMMØ derived from WT mice and mice deficient in both cathepsin S and L (Cat S-/-L-/-) were examined for their ability to activate MOG35-55-specific CD4+ T cells following incubation with MOG35-55 peptide (0, 10, 25 μg/ml) or MOG1-125 (0, 10, 25 μg/ml). Activation of MOG35-55-specific 2D2 CD4+ T cells was determined by surface expression of CD69 after exposure to the pulsed and washed BMMØs. Data represent 4 independent experiments. Data presented as mean+/- SEM; significant differences (unpaired students t-test, p<0.05) from the WT internal control are denoted by asterisks (*).
