# Supplementary material for: Redundancy between Cysteine Cathepsins in Murine Experimental Autoimmune Encephalomyelitis
Source: PLoS One. 2015 Jun 15;10(6):e0128945. doi: 10.1371/journal.pone.0128945 (PMC4468166; doi:10.1371/journal.pone.0128945)
Supplement: S1 Protocol — Methods for RNA extraction, cDNA synthesis, and QPCR of cathepsin mRNA levels in WT and cathepsin deficient BMMØs shown in S1 Fig. (DOCX) [file pone.0128945.s009.docx]

**S1 Protocol. RNA extraction, cDNA synthesis and real-time polymerase chain reaction (QPCR).** Methods for RNA extraction, cDNA synthesis, and QPCR of cathepsin mRNA levels in WT and cathepsin deficient BMMØs shown in S1 Fig.

RNA was extracted from WT, cathepsin B (Cat B^-/-^), cathepsin S (Cat S^-/-^), and cathepsin L (Cat L^-/-^) deficient BMMØ following the procedure outlined in the Aurum Total mRNA Mini Kit (BioRad). cDNA was made from 0.4 μg of RNA as outlined in the iScript Reverse Transcription Supermix for RT-qPCR (BioRad) [[1](#_ENREF_1)]. All primers were: at 300 nM; had a single melt curve; had efficiencies between 90-100%; and were designed or verified using Primer 3 (NCBI). Primers all resulted in one amplicon (as determined by melt-curves produced) and had between 85% and 105% efficiency. 18S was used as an internal control, did not vary across treatments. A BioRad iQ5 real-time thermal cycler was used to analyze gene expression (BioRad). Samples were set up according to the iQ SYBER Green Supermix (BioRad) protocol. Primers for cathepsin B (FWD 5’-GAAGAAGCTGTGTGGCACTG-3’REV 5’-GTTCGGTCAGAAATGGCTTC-3’), cathepsin S (FWD: 5’- TTCTTGTGGTGCCTGCTGGGC-3’REV: 5’- TGTAGCCGCCTCCACAGCCTT-3’), and cathepsin L (FWD 5’-ATGGCACGAATGAGGAAGAG-3’ REV 5’-GAAAAAGCCTCCCCTTCTTG-3’) [[2](#_ENREF_2)] were used with the following PCR conditions: 95° for 15 min; 40 cycles at 95° degrees for 60 sec, 55° for 60 sec. 18S (FWD: 5’- **A**GTCGGCATCGTTTATGGTC-3’REV: 5’-CGCGGTTCTATTTTGTTGGT-3’) was used as an internal control, and did not vary across treatments, with the following PCR conditions: 95° for 15 min; 40 cycles of 95° for 30 sec, 58° for 30 sec [[1](#_ENREF_1)]. Expression is presented relative to 18S and relative to the mock control samples.

1. Balce DR, Li B, Allan ER, Rybicka JM, Krohn RM, et al. (2011) Alternative activation of macrophages by IL-4 enhances the proteolytic capacity of their phagosomes through synergistic mechanisms. Blood 118: 4199-4208.

2. Dong Z, Katar M, Linebaugh BE, Sloane BF, Berk RS (2001) Expression of cathepsins B, D and L in mouse corneas infected with Pseudomonas aeruginosa. Eur J Biochem 268: 6408-6416.
